# The transcriptomic profile of peripheral blood nuclear cells in dogs with heart failure

**DOI:** 10.1186/1471-2164-15-509

**Published:** 2014-06-21

**Authors:** Magdalena Hulanicka, Magdalena Garncarz, Marta Parzeniecka-Jaworska, Michał Jank

**Affiliations:** Department of Physiological Sciences, Faculty of Veterinary Medicine, Warsaw University of Life Sciences, Nowoursynowska str. 159c, 02-776 Warsaw, Poland; Department of Veterinary Diagnostics and Pathology, Faculty of Veterinary Medicine, Warsaw University of Life Sciences, Nowoursynowska str. 159c, 02-776 Warsaw, Poland

**Keywords:** Dogs, Heart failure, ISACHC, Transcriptomic profile, Microarrays

## Abstract

**Background:**

In recent years advances have been made in the investigative methods of molecular background of canine heart disease. Studies have been conducted to identify specific genes which, when pathologically expressed, could lead to the dysfunction of the canine heart or are correlated with heart failure. For this purpose genome wide microarray experiments on tissues from failing hearts have been performed. In the presented study a whole genome microarray analysis was used for the first time to describe the transcription profile of peripheral blood nuclear cells in dogs with heart failure. Dogs with recognized heart disease were classified according the ISACHC (International Small Animal Cardiac Health Council) classification scheme as class 1 (asymptomatic) - 13 dogs, class 2 (mild to moderate heart failure) - 13 dogs and class 3 (severe heart failure) - 12 dogs. The control group consisted of 14 healthy dogs. The clinical picture of the animals included: animal history, clinical examination, echocardiographic examination and where applicable electrocardiographic and radiographic examinations.

**Results:**

In the present study we identified four sets of differentially expressed genes, namely heart-failure-specific genes and ISACHC1-specific genes, ISACHC2-sepcific genes and ISACHC-3 specific genes. The most important set consisted of genes differentially expressed in all dogs with heart failure, despite the ISACHC stage. We identified 71 heart-failure-specific genes which were involved in two statistically significant receptor signalling pathways, namely angiotensinR - > CREB/ELK-SRF/TP53 signalling and ephrinR - > actin signalling. The number of ISACHC1-specific genes was 83; ISACHC2-specific genes - 1247 and ISACHC3-specific - 200.

**Conclusions:**

The transcriptomic profile of peripheral blood nuclear cells in dogs with heart failure seems to reflect the presence of clinical signs of the disease in patients based on the observation that the largest number of differentially expressed genes was identified in ISACHC 2 group of patients. This group consists of dogs just starting to show clinical signs of heart failure. A set of genes was also found to have changed expression in all dogs with heart failure, despite the stage of the disease.

**Electronic supplementary material:**

The online version of this article (doi:10.1186/1471-2164-15-509) contains supplementary material, which is available to authorized users.

## Background

In recent years some advances have been made in the methods of investigation of the molecular background of canine heart disease. The etiopathogenesis of one of the most frequent heart diseases, myxomatous valve disease, in dogs may be different compared to humans although some similarities have been noted [1]. Some research has been conducted to identify specific genes which, when pathologically expressed, could lead to the dysfunction of the canine heart or are correlated with heart failure. For this purpose genome wide microarray experiments on tissues from failing hearts have been performed. Their results revealed that expression of some genes differ between tissue samples from failing hearts and healthy hearts, including: the matrix metalloproteinases and their tissue inhibitors (mainly *MMP9*, *MMP1*, *MMP2*, *TIMP1*, *TIMP3*) as well as genes involved in Ca^2+^ handling in cardiomyocytes, e.g. genes encoding the components of the cardiac ryanodyne receptor (RyR2) [2–6]. Other examples of differentially expressed genes are TGF-β (*TGF*-*βR2*, *TGF*-*βR3*) and serotonin receptors (*5HT*-*R2B*) [2]. The genome pattern of heart tissues from dogs suffering from heart failure were also investigated with the Real-time PCR method and the level of proteins encoded by differentially expressed genes was examined by immunohistochemical techniques. These findings gave similar results and pointed out different expression of extracellular matrix (ECM) genes, TGF-β receptors and thyroid hormone receptors (TRβ1 and TRβ2) in failing hearts compared to healthy control tissues [7–12].

The most important obstacle of the mentioned types of studies is access to diagnostic material. That is why researchers are attempting to investigate gene expression of peripheral blood nuclear cells taken from patients with cardiac disease. Blood nuclear cells are not a perfect source of diagnostic material in patients with heart failure but easy access makes them a very interesting source of potential disease markers. It has turned out that identification of such markers is possible since Wang et al. described the prognostic value of B-natriuretic peptide (BNP) in humans [13]. The results of a few studies suggest that expression of Na + −Ca2+ exchanger (*NCX*-*1*) gene in peripheral blood nuclear cells could have prognostic or diagnostic value [14, 15]. The other genes proposed as genomic markers in dogs are phospholamban (*PLN*) and haematopoietic lineage cell-specific protein 1 associated protein X-1 (*HAX*-*1*). Fonfara et al. also pointed out that the blood cells of dogs with heart failure could be appropriate material for searching for genomic markers. Their results showed increased levels of proinflammatory cytokines (*Il*-*1*, −*2*, −*8*, *INF*), *MMP*-*1*, *MMP*-*3* and *TIMP*-*3* and decreased levels of *TNF*-*10*, *Il*-*10*, *TGF*-*b1*, *TIMP*-*1* and *TIMP*-*2* in dogs with congestive heart failure [16].

In the present paper a whole genome microarray analysis was used for the first time to describe the transcription profile of peripheral blood nuclear cells in dogs with heart failure. The potential identification of any prognostic marker of canine cardiac disease would be of great importance since it may not only give new insight into the pathogenesis or staging of the disease but also help in the effective planning of treatment.

## Results

### The number of differentially expressed genes

Comparison of gene expression between all study groups (healthy dogs, ISACHC 1, ISACHC 2, ISACHC 3) revealed 4579 differentially expressed transcripts. Using the Tukey's HSD Post-hoc test it was possible to identify differentially expressed transcripts between individual study groups (Table 1).

**Table 1:** **The number of differentially expressed genes in dogs with different ISACHC stages of heart disease**

Name of group	ISACHC 2	ISACHC 1	Healthy dogs
ISACHC 3	2140	696	1170
ISACHC 2	-	3499	2964
ISACHC 1	-	-	426

### Heart failure specific and ISACHC stage specific genes

The next step of our analysis was to identify heart-failure-specific genes. This means genes which were differentially expressed in all dogs with heart disease, despite the stage of the disease. The comparison between healthy and ISACHC 1 group revealed 426 differentially expressed transcripts; between healthy and ISACHC 2 dogs revealed 2964 differentially expressed transcripts and between healthy and ISACHC 3 dogs revealed 1170 differentially expressed transcripts. Among these three sets of differentially expressed genes there were 117 common transcripts. However, since some genes are represented on the array by more than one spot, we verified the consistency of differential expression in all the spots representing the same genes to eliminate false positive results. Moreover, the ontologies of all the transcripts are not known. Taking into consideration these two facts, a list of 71 heart - failure - specific genes of known ontologies was obtained (Table 2). The direction of changes in expression (up or down) of all 71 genes was the same in each disease stage. If a gene was down-regulated, this means that it was down regulated in all three comparisons (healthy vs ISACHC 1; healthy vs ISACHC 2; healthy vs ISACHC 3).

**Table 2:** **The list of the differentially regulated heart**-**failure**-**specific genes in dogs with ISACHC heart failure**

GeneSymbol	Adjusted p-values	Regulation	Description
*ACTA1*	0.014573121	Up	Actin, alpha 1, skeletal muscle
*ACTA2*	0.039442863	Up	Actin, alpha 2, smooth muscle, aorta
*ACTC1*	0.024042822	Up	Actin, alpha, cardiac muscle 1
*ACTL7A*	0.024659734	Up	Actin-like 7A
*ADA*	0.01982955	Up	Adenosine deaminase F
*ATF4*	0.044055864	Up	Activating transcription factor 4
*CAMP*	0.018274972	Up	Cathelicidin antimicrobial peptide
*CAPG*	0.046687745	Up	Capping protein (actin filament), gelsolin-like
*CCL14*	0.024452273	Up	Chemokine (C-C motif) ligand 14
*CD151*	0.012394691	Up	CD151 molecule (Raph blood group)
*CD177*	0.010730711	Up	CD177 molecule
*CD300E*	0.0064891023	Up	CD300e molecule
*CORO1B*	0.03894925	Up	Coronin, actin binding protein, 1B
*CRYL1*	0.0078813145	Up	Crystallin, lambda 1
*CYBA*	0.031601883	Up	Cytochrome b-245, alpha polypeptide
*CYP27A1*	0.034636706	Up	Cytochrome P450, family 27, subfamily A, polypeptide 1
*DPEP2*	0.008431375	Up	Dipeptidase 2
*DSTN*	0.008163466	Up	Destrin (actin depolymerizing factor)
*EAF1*	0.03957692	Up	ELL associated factor 1
*EPHX2*	0.018156866	Up	Epoxide hydrolase 2, cytoplasmic
*FAM107A*	0.020942843	Up	Family with sequence similarity 107, member A
*FANCG*	0.0043643974	Down	Fanconi anemia, complementation group G
*FLOT2*	0.017366651	Up	Flotillin 2
*GP9*	0.0075741783	Up	Glycoprotein IX (platelet)
*GSN*	0.006870092	Up	Gelsolin
*HK3*	0.0073725334	Up	Hexokinase 3 (white cell)
*HP*	0.016630936	Up	Haptoglobin
*HPCAL1*	0.030948874	Up	Hippocalcin-like 1
*IL10RA*	0.046400532	Up	Interleukin 10 receptor, alpha
*KDELR1*	0.0037026592	Up	KDEL (Lys-Asp-Glu-Leu) endoplasmic reticulum protein retention receptor 1
*KLHDC8B*	0.015807072	Up	Kelch domain containing 8B
*LOC475113*	0.008475645	Up	Lymphocyte antigen 6B-like
*LOC491090*	0.027546933	Up	Similar to tubulin, alpha 1
*LSM10*	0.0052581653	Up	LSM10, U7 small nuclear RNA associated
*MAPK1*	0.03080859	Up	Mitogen-activated protein kinase 1
*ME1*	0.016186723	Up	Malic enzyme 1, NADP(+)-dependent, cytosolic
*MMP8*	0.008103578	Up	Matrix metallopeptidase 8 (neutrophil collagenase)
*MMRN1*	0.005678207	Up	Multimerin 1
*MOV10L1*	0.012869541	Up	Mov10l1, Moloney leukemia virus 10-like 1, homolog (mouse)
*MRPL37*	0.026399544	Up	Mitochondrial ribosomal protein L37
*MVP*	0.008610116	Up	Major vault protein
*MYL6*	0.032878894	Up	Myosin, light chain 6, alkali, smooth muscle and non-muscle
*MYL6B*	0.008475645	Up	Myosin, light chain 6B, alkali, smooth muscle and non-muscle
*N4BP2*	0.01824313	Down	NEDD4 binding protein 2
*NAGPA*	0.038744777	Up	N-acetylglucosamine-1-phosphodiester alpha-N-acetylglucosaminidase
*NEIL1*	0.030278882	Down	Nei endonuclease VIII-like 1 (E. coli)
*NQO2*	0.037860617	Up	NAD(P)H dehydrogenase, quinone 2
*NRGN*	0.014320144	Up	Neurogranin (protein kinase C substrate, RC3)
*NSG1*	0.009202879	Up	Neuron specific gene family member 1
*PARVB*	0.025306692	Up	Parvin, beta
*PCGF2*	0.015807072	Up	Polycomb group ring finger 2
*PGLYRP1*	0.0151561815	Up	Peptidoglycan recognition protein 1
*PPP1R3D*	0.0077612605	Up	Protein phosphatase 1, regulatory (inhibitor) subunit 3D
*PTGER3*	0.0045230556	Up	Prostaglandin E receptor 3 (subtype EP3)
*PTPN9*	0.008163466	Up	Protein tyrosine phosphatase, non-receptor type 9
*RSU1*	0.046061113	Up	Ras suppressor protein 1
*SEPT5*	0.0062189046	Up	Septin 5
*SHC2*	0.02669537	Up	SHC (Src homology 2 domain containing) transforming protein 2
*SLC11A1*	0.0045230556	Up	Solute carrier family 11 (proton-coupled divalent metal ion transporters), member 1
*SLC27A2*	0.006745509	Up	Solute carrier family 27 (fatty acid transporter), member 2
*SLC2A6*	0.0045230556	Up	Solute carrier family 2 (facilitated glucose transporter), member 6
*TBC1D30*	0.030404283	Up	TBC1 domain family, member 30
*TBXAS1*	0.0049617672	Up	Thromboxane A synthase 1 (platelet)
*TCIRG1*	0.023556981	Up	T-cell, immune regulator 1, ATPase, H + transporting, lysosomal V0 subunit A3
*TIMP1*	0.0043643974	Up	TIMP metallopeptidase inhibitor 1
*TOP3B*	0.02519754	Up	Topoisomerase (DNA) III beta
*TRPV2*	0.0049617672	Up	Transient receptor potential cation channel, subfamily V, member 2
*TSPO*	0.004600876	Up	Translocator protein (18 kDa)
*TTC19*	0.0075741783	Down	Tetratricopeptide repeat domain 19
*VNN1*	0.031601883	Down	Vanin 1
*ZNF248*	0.004831977	Down	Zinc finger protein 248

After identifying heart - failure - specific genes we applied the same approach to a selection of each stage - specific genes. The 83 genes specific for ISACHC class 1 were identified (data not shown). The list of ISACHC class 2 - specific genes was the longest and consisted on 1247 genes (data not shown). In cases of most advanced disease, ISACHC 3, the list of specific genes was 200 (data not shown). These four lists of genes were subjected to further analysis.

### Pathways connected with differentially expressed genes

Next we examined the differentially regulated genes for enrichment in any known canonical pathway. For this purpose the Pathway Studio software was used. The most interesting results were obtained from analysis of the Ariadne Receptor Signaling Pathways. The results of this analysis are presented in Tables 3, 4, 5, 6.

**Table 3:** **Ariadne pathways based on heart**-**failure**-**specific genes differentially regulated in dogs with ISACHC heart failure**

Ariadne receptor signaling pathways	The genes engaged in listed pathways	p-value
EphrinR - > actin signaling	*ACTA2*, *GSN*, *ACTA1*, *ACTC1*, *DSTN*	0.0179871
AngiotensinR - > CREB/ELK-SRF/TP53 signaling	*MAPK1*, *CYBA*, *NQO2*	0.0359593

**Table 4:** **Ariadne pathways based on ISACHC1**-**specific genes differentially regulated in dogs with ISACHC1 heart failure**

Ariadne receptor signaling pathways	The genes engaged in listed pathways	p-value
PTPRC - > BCL6 signaling	2	0.0221563
BradykininR - > STAT3 signaling	1	0.0349728
TNFR - > AP-1/ATF/TP53 signaling	2	0.0462323

**Table 5:** **Ariadne pathways based on ISACHC2**-**specific genes differentially regulated in dogs with ISACHC2 heart failure**

Ariadne receptor signaling pathways	The number of genes	p-value
UrokinaseR - > ELK-SRF signaling	9	0.0010517
IL4R - > ELK-SRF/HMGY signaling	9	0.00118083
PDGFR - > FOXO3A signaling	5	0.00176524
IL2R - > ELK-SRF/MYC signaling	8	0.00234544
AGER - > NF-kB signaling	6	0.00241572
IL15R - > NF-kB/NFATC signaling	6	0.0028208
IL7R - > FOXO/NF-kB signaling	6	0.00327501
InsulinR - > CTNNB/FOXA/FOXO signaling	5	0.0038699
FibronectinR - > CTNNB signaling	6	0.00434523
TNFRSF5/13B - > NFATC1 signaling	5	0.00459712
VEGFR - > CTNNB signaling	5	0.00459712
PTPRC - > STAT6 signaling	2	0.00520887
FGFR - > AP-1/CREB/CREBBP/ELK-SRF/MYC signaling	10	0.00699557
HGFR - > FOXO3A signaling	4	0.00709744
AdenosineR - > NF-kB signaling	7	0.0071087
ErythropoietinR - > FOXO3A signaling	4	0.00859443
TLR3 - > NF-kB signaling	3	0.00948063
ICAM2 - > CTNNB/FOXO/STAT3 signaling	5	0.00974526
CD19 - > NF-kB signaling	5	0.00974526
InsulinR - > ELK-SRF/SREBF signaling	7	0.0104641
FibronectinR - > AP-1/ELK-SRF/SREBF signaling	10	0.0106044
Notch - > NF-kB signaling	5	0.0111183
GHR - > NF-kB signaling	5	0.0111183
IL12R - > NF-kB/NFATC signaling	5	0.0126187
DopamineR2 - > NF-kB signaling	5	0.0126187
ThrombinR - > NF-kB signaling	5	0.0126187
ErythropoietinR - > NF-kB signaling	6	0.0138912
AGER - > CREB/SP1 signaling	5	0.0142517
AngiopoietinR - > FOXO signaling	4	0.0142826
VEGFR - > FOXO3A signaling	4	0.0166053
CholinergicRm - > CREB/ELK-SRF signaling	10	0.0174704
B-cell receptor - > NFATC signaling	5	0.0179347
ErythropoietinR - > AP-1/CREB/MYC signaling	8	0.0182859
EDG2 - > ELK-SRF signaling	7	0.0189356
SerotoninR1 - > FOS signaling	10	0.019617
HGFR - > AP-1/CREB/MYC signaling	8	0.0209776
IGF1R - > CEBPA/FOXO1A signaling	4	0.0219319
FcIgER - > ELK-SRF signaling	7	0.0220582
TLR1/2/6 - > NF-kB signaling	5	0.0222033
DopamineR2 - > AP-1/CREB/ELK-SRF signaling	9	0.024631
GDNF - > HSF1 signaling	7	0.025526
ThrombopoietinR - > AP-1/CREB/ELK-SRF/MYC signaling	8	0.0255337
VEGFR - > AP-1/CREB/MYC signaling	8	0.0289362
MacrophageR - > CEBPB/NF-kB signaling	5	0.0326186
TLR3 - > IRF signaling	2	0.0326546
FibronectinR - > NF-kB signaling	6	0.032985
GHR - > ELK-SRF/MYC signaling	6	0.0355346
B-cell receptor - > NF-kB signaling	6	0.0382096
CholinergicRn - > CREB signaling	5	0.0388136
NTRK - > FOXO/MYCN signaling	4	0.0394373
EGFR - > AP-1/CREB/ELK-SRF/MYC signaling	8	0.0410289
IL6R - > STAT signaling	2	0.0423644
PDGFR - > AP-1/MYC signaling	7	0.043159
CD38 - > NF-kB signaling	7	0.043159
KIT - > MITF signaling	6	0.043943
NTRK - > AP-1/CREB/ELK-SRF/MYC/SMAD3/TP53 signaling	8	0.0457237
PTAFR - > AP-1/ATF1/CREB/ERK-SRF signaling	9	0.0466592
IGF1R - > MEF/MYOD/MYOG signaling	6	0.0470043
CD19 - > AP-1/ELK-SRF signaling	6	0.0470043
PTAFR - > NF-kB signaling	6	0.0470043
ThrombopoietinR - > SPI1 signaling	4	0.0481701

**Table 6:** **Ariadne pathways based on ISACHC3**-**specific genes differentially regulated in dogs with ISACHC3 heart failure**

Ariadne receptor signaling pathways	The number of genes	p-value
HGFR - > FOXO3A signaling	3	0.00563574
ERBB2/3 - > EP300/ETS/ETV/SP1 signaling	5	0.01015
EGFR/ERBB2 - > HIF1A signaling	5	0.0128385
TNFRSF5/13B - > NFATC1 signaling	3	0.0152928
HGFR - > AP-1/CREB/MYC signaling	5	0.0216208
AdenosineR - > NF-kB signaling	4	0.0217062
ICAM2 - > CTNNB/FOXO/STAT3 signaling	3	0.0242659
NeurotensinR - > ELK-SRF/AP-1/EGR signaling	5	0.0248821
IL2R - > ELK-SRF/MYC signaling	4	0.0259176
InsulinR - > ELK-SRF/SREBF signaling	4	0.0274252
GHR - > ELK-SRF/MYC signaling	4	0.0274252
KIT - > MITF signaling	4	0.032265
IGF1R - > MEF/MYOD/MYOG signaling	4	0.033985
UrokinaseR - > ELK-SRF signaling	4	0.0357589
IL4R - > ELK-SRF/HMGY signaling	4	0.0375869
FibronectinR - > CTNNB signaling	3	0.0381165
EDG2 - > ELK-SRF signaling	4	0.0394692
EGFR/ERBB3 - > MEF/MYOD/NFATC/MYOG signaling	6	0.0414187
FcIgER - > ELK-SRF signaling	4	0.0433973
EGFR - > NCOR2 signaling	4	0.0454432
GDNF - > HSF1 signaling	4	0.047544
ErythropoietinR - > ELK-SRF/FOS signaling	5	0.0477064

### Real-time RT-PCR

**Figure 1:**
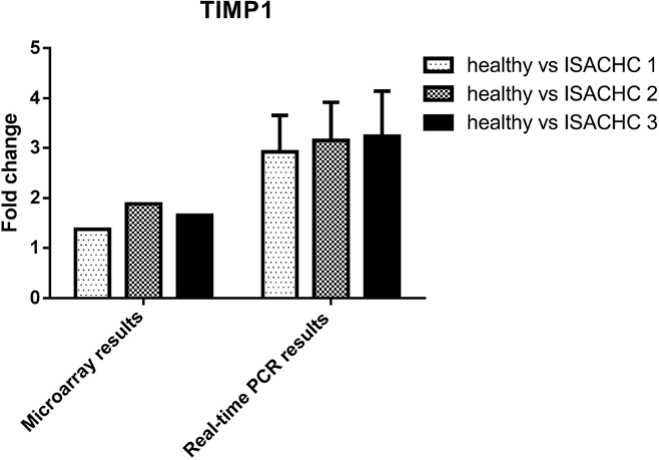
**The expression TIMP1 gene measured with microarray and Real-time PCR in dogs with different ISACHC stages of heart disease.**

**Figure 2:**
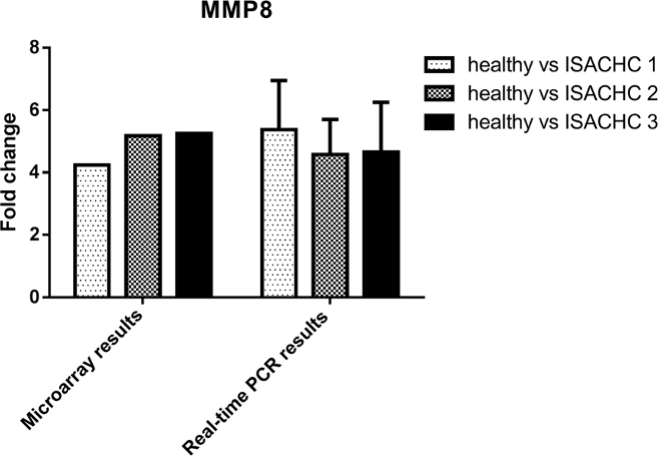
**The expression MMP8 gene measured with microarray and Real-time PCR in dogs with different ISACHC stages of heart disease.**

**Figure 3:**
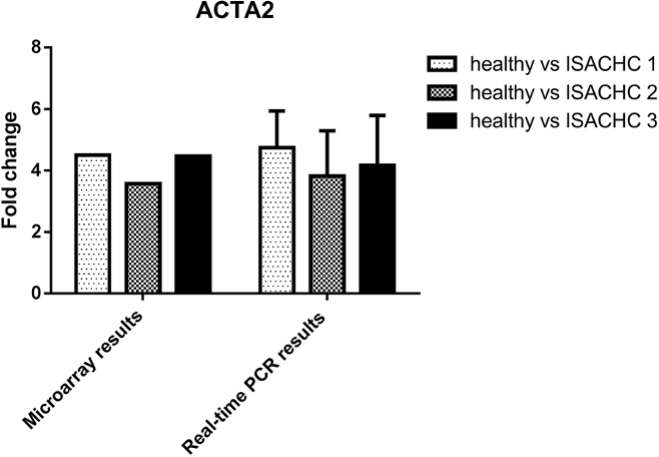
**The expression ACTA2 gene measured with microarray and Real-time PCR in dogs with different ISACHC stages of heart disease.**

To validate microarray data we selected three genes for Real-time PCR analysis (*ACTA2*, *TIMP1*, *MMP8*). The changes in the expression of these three genes measured using Real-time qPCR were similar to gene expression changes observed in the microarray studies (Figures 1, 2, 3).

## Discussion

Heart failure in dogs does not occur as frequently as it does in humans and is mainly caused by two diseases, namely endocardiosis (also referred to as myxomatous valve disease or chronic degenerative valvular disease) and dilated cardiomyopathy. Both these diseases can lead to congestive heart failure and the associated clinical signs. Since the molecular background of these diseases, especially endocardiosis, is still far from fully elucidated, the search for any specific markers (prognostic, therapeutic) of this disease would be of great value.

In the present study we evaluated the transcriptomic profile of peripheral blood nuclear cells of dogs with different stages of heart disease. Since the whole analysis resulted in the huge number of differentially expressed transcripts (4579) we decided to identify two types of genes - 1) genes differentially expressed in all affected dogs, despite the ISACHC stage (so called heart-failure-specific genes) and 2) genes differentially expressed in each of ISACHC stages (so called ISACHC stage-specific genes). This approach seems reasonable because if the gene is regulated in all patients with heart failure, despite the stage of the disease, it could be a potential marker of heart disease in dogs. On the other hand the identification of each ISACHC stage-specific gene give the opportunity to identify markers of heart disease progression (i.e. form stage I to stage II or from stage II to stage III).

The list of heart-failure specific genes included 117 transcripts for which we were able to identify 71 specific genes. Among these genes we were able to identify three α-SMA genes which are considered as a markers of muscle cells. This phenomenon could suggest the presence of myofibroblasts (circulating fibroblasts) in the peripheral blood nuclear cells of dogs with heart failure. Similar observation has been recently published in humans with atrial fibrillation what could indicate that heart disease could be related with the presence of α-SMA containing cells in the blood [17]. However, α-SMA containing cells could also be of hematopoietic progenitor cells origin so their source in the blood of heart disease patients is still unclear [18]. Moreover, our results of flow cytometer analysis showed that the percentage of α-SMA positive cells in peripheral blood mononuclear cells of three dogs with ISACHC class 2 heart failure was between 0.2-0.5% (Additional files 1, 2). Whether these cells could be a marker of heart disease remains a pure speculation. Other genes present among 71 heart failure specific genes have been previously related to heart failure or involved in heart disease development. Examples include matrix metallopeptidase 8 (*MMP8*) and TIMP metallopeptidase inhibitor 1 (*TIMP1*). It is known that remodelling of the extracellular matrix (ECM) occurs during heart failure and the matrix metalloproteinases and their tissue inhibitors are involved in this process [19]. Initially *MMP*-*8* was considered to be only expressed in peripheral inflammatory cells, but its myocardial sources could be endogenous cell types such as mast cells [20]. The level of *MMP8* is increased during left ventricular remodelling following myocardial infarction in rats [21]. Our study showed that the expression of *MMP8* is increased in all study groups in comparison to healthy control dogs (over a 4 fold change). Moreover, these results were confirmed with Real-time PCR (Figure 2). The study conducted by Oyama and Chittur revealed that the expression of *TIMP1* was over 4 times higher in dogs with endocardiosis than in healthy dogs [4]. Our results (both microarray and Real-time PCR) showed a similar tendency. Another gene identified as heart - failure - specific one was haptoglobin (*HP*). The expression of *HP* was higher (over 2.4 fold change) in diseased dogs than in control dogs. In humans the functional allelic polymorphism in the *HP* gene plays a key role in determining survival and presence of congestive heart failure after myocardial infarction [22]. However, it seems quite impossible that single gene expression could influence the complex processes of heart disease development. Rather relationships and interactions between differentially expressed genes could play a key role. In order to evaluate these possibilities we used the Pathway Studio software. This database gives the opportunity to find genes involved in different kinds of signalling pathways. Pathway Studio software can also group differentially expressed genes on the basis of their molecular function and biological processes in which they are involved. The genes differentially expressed with statistical significance identified as heart-failure-specific were involved in regulation of two canonical pathways, namely angiotensinR - > CREB/ELK-SRF/TP53 signalling and ephrinR - > actin signalling. The first signalling pathway shows the relation between the expression of angiotensin receptors and CREB/ELK-SRF/TP53 transcription factors and indicates that the combined activation of CREB/ELK-SRF/TP53 leads to angiotensin receptor activation during the disease process. Serum response factor (*SRF*) is a transcription factor which regulates many immediate-early genes and is required for the appearance of beating sarcomeres in the heart [23]. *SRF* activates genes involved in smooth muscle differentiation and proliferation by recruiting muscle-restricted cofactors, such as ternary complex factors (TCFs) of the ETS-domain family, for example *ELK*-*1* [24]. Changed expression of *ELK*-*4* in peripheral blood nuclear cells in patients with refractory ischemic end-stage heart failure were also seen in our previous study [25] and Kuner et al. recognized *ELK*-*4* as potential interaction partner necessary for creating network of regulatory genes in ischemic cardiomyopathy [26]. Since CREB/ELK-SRF/TP53 transcription factors target angiotensin receptors in dogs with heart failure, it clearly proves that the function of the failing heart is directly correlated with renal function and that in cases of heart disease we could observe changes in the function of renin-angiotensin-aldosterone axis. Such a conclusion was also drawn from one of our previous studies in which we were able to identify that transcriptomic profile of peripheral blood nuclear cells in humans after acute heart failure is correlated with the creatinine (marker of kidney function) level in the blood [27].

Since the lists of genes specific for each heart failure stage were much longer than the list of heart-failure-specific genes we also ran the Pathway Studio analysis to obtain information on significantly regulated signalling pathways in each ISACHC stage (Tables 4, 5, 6). While analysing these results one has to remember that ISACHC 1 heart failure stage encompasses patients without clinical signs of heart disease, ISACHC 2 stage includes dogs with compensated clinical signs and ISACHC 3 stage includes dogs with uncompensated heart failure. Having this in mind one could conclude the very long list of differentially regulated genes as well as significantly regulated signalling pathways in ISACHC 2 patients is not only the results of heart disease but also the result of many different compensatory mechanisms which were triggered in order to restore homeostasis. On the other hand in ISACHC 3 patients, the majority of these mechanisms are no longer in place so the list of regulated genes and pathways is much shorter. Based on our results it could be concluded that for the ISACHC 1 group, the specific changes in the transcriptome include the regulation of protein tyrosine phosphatase kinase receptors, bradykinin receptor and tumor necrosis factor receptor. These changes seem to be rather unspecific for heart disease and probably reflect only the reaction of the body to progressed disease, not the progression of the disease itself. In case of ISACHC 2 specific genes and pathways the interpretation of the obtained results is complex but in our opinion it is worth to point out that in addition to the statistically significant regulation of many inflammatory pathways and growth factors, we also see changes in the regulation of transcription factors targeted at platelet-mediated mediators and *PTAFR* (platelet-activating factor receptor). Anti-platelet therapy is one of the standard approaches in the treatment of patients with heart failure in humans and the regulation of this receptor was also identified in our previous study on patients with end-stage ischemic heart failure [25]. Platelet-activating factor may play a critical role in the development of severe heart failure coupled with resulting multiple organ failure, as seen in the Munakata et al. study performed on dogs with left ventricular assistance post coronary ligation [28]. This study showed that it may also be beneficial for dogs with clinical signs of severe heart disease to receive some form of anti-platelet therapy.

In the pathways significantly regulated in ISACHC 3 patients, we were able to identify for the first time the presence of transcription factors targeting EGFR/ERBB2. The ErbB2(HER2)-dependent signalling pathway is involved in proper myocardium function, structure and contractility [29–31]. The ErbB2 (*HER2*) receptor is responsible for the activation of several transcription factors, including *AP*-*1* (also in heart muscle hypertrophy) and nuclear factor kappa B (involved in the response to oxidative stress) [32]. It could play a key role in dilated cardiomyopathy prevention [33]. In mice with *ErbB2* gene deletion a gradual development of systolic myocardial dysfunction has been observed, occurring with myocardium thinning and heart ventricles distension [34]. In mice the binding of neuregulin-1 (*NRG*-*1*) to the *HER2* receptor is cardioprotective [35]. Since there is no data in the literature in this field obtained from dogs, we could only speculate that the regulation of ErbB2 signalling pathway in dogs with ISACHC 3 heart failure could be the feature of end-stage heart failure also in this species. But this observation requires further investigation.

## Conclusions

The transcriptomic profile of peripheral blood nuclear cells in dogs with heart failure seems to reflect the presence of clinical signs of the disease in patients. Such a conclusion could be drawn from the observation that the longest list of differentially expressed genes as well as signalling pathways were identified in ISACHC 2 group of patients, those first to show clinical signs of heart failure. The development of heart failure in dogs results in changed expression of genes involved in the regulation of the renin-angiotensin-aldosterone axis in peripheral blood nuclear cells whereas end-stage heart failure could be reflected by activation of transcription factors targeting at the ErbB2 receptor.

## Methods

### Animals

This study complies with national and institutional guidelines on the use of animals in clinical research according to the Polish legal act from January 21st, 2005 (Ustawa o doświadczeniach na zwierzętach z dnia 21 stycznia 2005 r. (Dz. U. z 2005 r. Nr 33, poz. 289 z późn.zm.)), concerning experiments performed on client owned animals. Before enrolling a dog into the study an informed consent from its owner was obtained and a high standard of care was adhered to throughout each examination. A prospective analysis and studies were carried out on dogs submitted to the Cardiology Service of the Small Animal Clinic, Faculty of Veterinary Medicine, School of Agriculture in Warsaw. The clinical picture of the animals included: animal history, clinical examination, echocardiographic examination and where applicable electrocardiographic and radiographic examinations. Dogs with recognized heart disease were classified according the ISACHC (International Small Animal Cardiac Health Council) classification scheme as class 1 (asymptomatic) - 13 dogs, class 2 (mild to moderate heart failure) - 13 dogs and class 3 (severe heart failure) - 12 dogs. The control group consisted of 14 healthy dogs. Study population characteristics are shown in Table 7. Statistical analyses of clinical parameters were performed using GraphPad Prism version 5.00 (GraphPad Software, Inc., USA).

**Table 7:** **Study population characteristics (data are shown as mean** ± **SD)**

ISACHC		Healthy	ISACHC 1	ISACHC 2	ISACHC 3
n = 52		14	13	13	12
Breeds		Dachshund (5), German Shepherds (6), Labrador retriever (1), German Pointer (1), Rottweiler (1)	Dachshund (10), Labrador retriever (1), Miniature poodle (1), Standard schnauzer (1)	Dachshund (9), Cocker spaniel (2), Airedale terrier (1), Standard schnauzer (1)	Dachshund (12)
Age range		1.4 - 10.5 years	5.75 - 14.0 years	9.8 - 15.2 years	7.25 - 15.2 years
Sex	Males	8	7	7	10
	Females	6	6	6	2
EF		66.08 ± 7.99	72.93 ± 9.33	77.0 ± 8,90**	83.08 ± 4.96***
HR		107.3 ± 18.90	113.7 ± 26.08	134.0 ± 21.29*	140.7 ± 19.43**

*Statistically significant difference vs healthy dogs with p<0.05; **statistically significant difference vs healthy dogs with p<0.01; ***statistically significant difference vs healthy dogs with p<0.001.

### Clinical and laboratory examinations

The clinical and laboratory examinations included: complete physical examination, including thoracic auscultation, assessment of dyspnoea, cough, heart rate, presence of heart murmurs, mucosal membrane colour, pulse, palpation of thorax and abdomen as well as additional cardiologic examinations: echocardiography, electrocardiography (I, II,III, aVL, aVF and aVR leads) in dogs with arrhythmias, radiography in dogs with suspicion of congestive heart failure, and blood pressure measurement (Doppler method). All of above mentioned examinations were carried out by one clinician from the Cardiology Service of Small Animal Clinic at Faculty of Veterinary Medicine. The blood morphology and biochemistry analyses were conducted in the Laboratory of Small Animal Clinic.

All examinations were performed at rest without pharmacological restraint. A transthoracic echocardiographic (TTE) examination was performed in all dogs with an Aloka 4000 ultrasound machine equipped with a cardiology programs and 2.5 - 7-megahertz (mHz) sector transducers. Electrocardiographic examination (leads I, II, III, aVL, aVF and aVR) were performed with a BTL-08 MD machine. Radiological examination of the chest were performed with the G&E Prestige II X-ray machine. The blood samples for the transcription profile analysis were collected from client-owned dogs during routine veterinary examinations.

### RNA isolation, validation, labelling, and hybridization

Blood samples were drawn from the cephalic or jugular veins and collected in Rneasy Protect Animal Blood Tubes (Qiagen, USA). Total RNA from peripheral blood nuclear cells was isolated using a Rneasy Protect Animal Blood Kit (Qiagen, USA). Isolated RNA samples were dissolved in 30 μl of REB Buffer from the test kit. Next the RNA quantity was measured spectrophotometrically using a NanoDrop (NanoDrop Technologies, USA). The analysis of final RNA quality and integrity was performed with a BioAnalyzer (Agilent, USA). To ensure optimal data quality, only RNA samples with RIN number ≥7.5 were included in the analysis.

The analysis of gene-expression profile was performed using Canine (V2) Gene Expression Microarray, 4x44K (Agilent Technologies, USA). Each slide contained 4 microarrays representing about 45000 canine predicted mRNAs. The Quick Amp Labeling Kit (Agilent, USA) was used to amplify and label target RNA to generate complementary RNA (cRNA) for oligo microarrays used in gene expression profiling. Experiment was performed using a common reference design, where the common reference was a pool of equal amounts of RNA from 10 healthy dogs. On each two-color microarrays, we hybridized 825 ng of cRNA from the pool (labelled Cy3) and 825 ng of cRNA (labelled Cy5). In total we ran 52 microarrays - one for each patient. Microarray hybridization was performed with the Gene Expression Hybridization Kit (Agilent Technologies, USA), according to the manufacturer's protocols. RNA Spike In Kit (Agilent Technologies, USA) was used as an internal control. Acquisition and analysis of hybridization intensities were performed using the Agilent DNA microarray scanner.

### Signal detection and statistical analysis

Data were extracted and background subtracted using the standard procedures contained in the Agilent Feature Extraction (FE) Software version 10.7.3.1. FE performs a Lowess normalization. The statistical analysis was performed using Gene Spring 12 software (Agilent, USA). The samples underwent quality control and the results showed that each sample had a similar QC metric profile. The next step was filtering probesets by flags to remove poor quality probes (absent flags). The statistical significance of the differences was evaluated using a one-way ANOVA and Tukey's HSD Post-hoc test (p < 0.05). A multiple testing correction was performed using Benjamini and Hochberg False Discovery Rate (FDR) < 5%. Microarray data were deposited at the Gene Expression Omnibus data repository under the number GSE48319 and followed MIAME requirements.

To identify signalling pathways and gene function the microarray data was analyzed using Pathway Studio 9.0 (Ariadne Genomics). This is a database consisting of millions of individually modelled relationships between proteins, genes, complexes, cells, tissues, drugs and diseases [36].

### Real-time RT-PCR

To verify microarray results on the expressions of *ACTA2* (the gene for the actin, alpha 2, smooth muscle, aorta), *MMP8* (the gene for the matrix metallopeptidase 8 (neutrophil collagenase)), *TIMP1* (the gene for the TIMP metallopeptidase inhibitor 1) were checked by real-time RT-PCR. The sequences of these genes were obtained from the Ensembl database. Primers were designed using Primer-Blast software (NCBI database) and then checked for secondary structures using Oligo Calculator (free on-line access). The secondary structures of the amplicon were examined using the mfold Web Server (free on-line access). *GAPDH* was used as a house keeping gene [37]. The sequences of the primers are listed in Table 8.

cDNA was synthesized using the Enhanced Avian HS RT-PCR Kit (Sigma-Aldrich, St. Louis, Missouri). All analyses were performed on individual samples of total RNA using a LightCycler FastStart DNA Master SYBR Green I kit (Roche Diagnostics GmBH, Germany) as follows: Mg^2+^ was added to a final concentration of 3 mM; pre-incubation at 95°C for 10 minutes; amplification (40 cycles) including denaturation at 95°C for 10 seconds, annealing – temperature and time specified in Table 8; melting curve including denaturation at 95°C for 0 seconds, annealing at 65°C for 15 seconds, continuous melting at 95°C for 0 seconds (slope = 0.1°C/s); cooling at 40°C for 30 seconds. Results were calculated using the 2^-ΔΔC^T method [38].

**Table 8:** **The sequences of the primers used in the study**

Primer	Forward	Reverse	Annealing conditions
ACTA2	GTGGGGATGGGACAAAAGG	GAAAGCACCGCCTGAATAG	60°C for 9 s
MMP8	CGATGCAGAAGAAACATGGA	TTGCTTGAAGGACCGTAGAT	60°C for 10 s
TIMP1	CTTAAACCGGCGTTATGAGA	GGGGATGGATGAACAGGTAA	60°C for 10 s
GAPDH	CTGGGGCTCACTTGAAAGG	CAAACATGGGGGCATCAG	59°C for 4 s

## Electronic supplementary material

Additional file 1: **Representative results of flow cytometric analysis showing negative control of immunofluorescence staining of peripheral blood mononuclear cells (PBMC) from dog number 1 with ISACHC 2 class heart failure.** PBMC were fixed, permeabilized and stained with secondary antibodies: chicken anti-mouse IgG conjugated with Alexa Fluor 488 (Molecular Probes, Life Technologies). The percentage of α-SMA positive cells is 0.0%; a) morphological flow cytometric parameters of analyzed cells gated on the basis of their size (forward scatter: FSC) and granularity (side scatter: SSC); b) cytogram of Alexa Fluor 488 positive cells (gate P2); c) histogram of Alexa Fluor 488 positive cells (gate P4); d) table showing population hierarchy of cells in cytograms a, b, and histogram c. (PDF 72 KB)

Additional file 2: **Representative results of flow cytometric analysis of peripheral blood mononuclear cells (PBMC) from dog number 1 with ISACHC 2 class heart failure, which were stained with antibodies against alpha smooth muscle actin (α-SMA).** PBMC were fixed, permeabilized and stained with monoclonal mouse anti- α-SMA antibodies (clone 1A4, Dako, Denmark), followed by incubation with secondary antibodies: chicken anti-mouse IgG conjugated with Alexa Fluor 488 (Molecular Probes, Life Technologies). The percentage of α-SMA positive cells is around 0.3%; a) morphological flow cytometric parameters of analyzed cells gated on the basis of their size (forward scatter: FSC) and granularity (side scatter: SSC); b) cytogram showing α-SMA –positive cells gated in gate P2; c) histogram showing α-SMA –positive cells gated in gate P4; d) table showing population hierarchy of cells in cytograms a, b, and histogram c. (PDF 85 KB)
