# Supplementary figures and images for: The transcriptomic profile of peripheral blood nuclear cells in dogs with heart failure
Source: BMC Genomics. 2014 Jun 21;15(1):509. doi: 10.1186/1471-2164-15-509 (PMC4092214; doi:10.1186/1471-2164-15-509)

a)

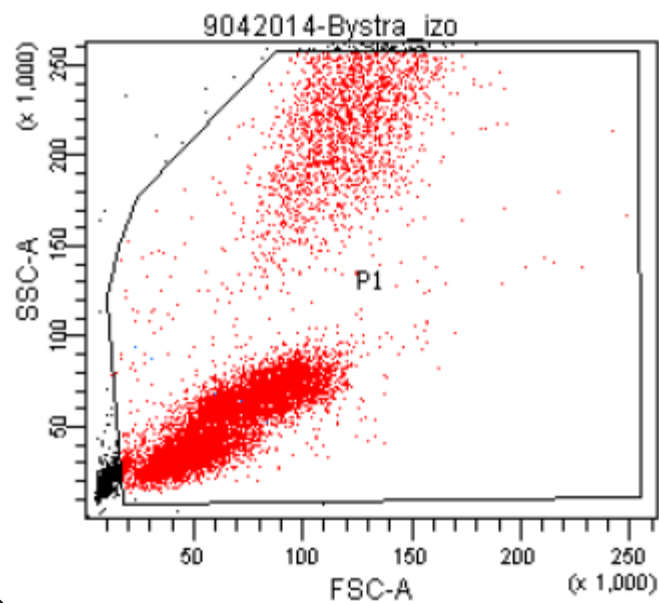

b)

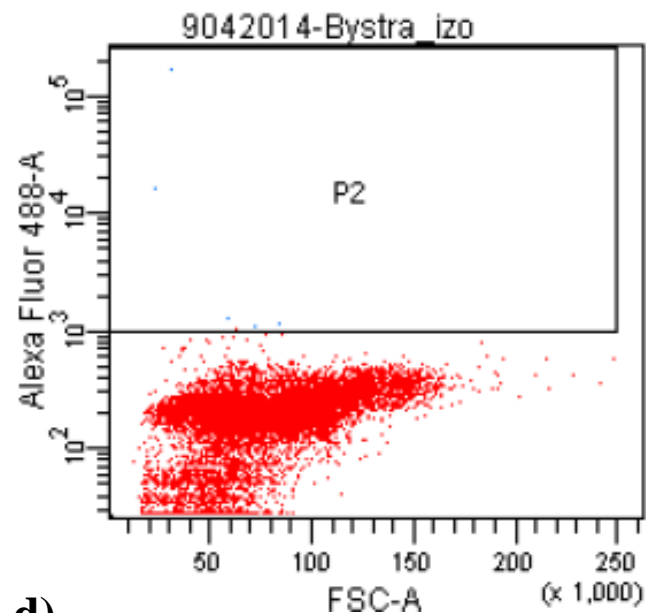

c)

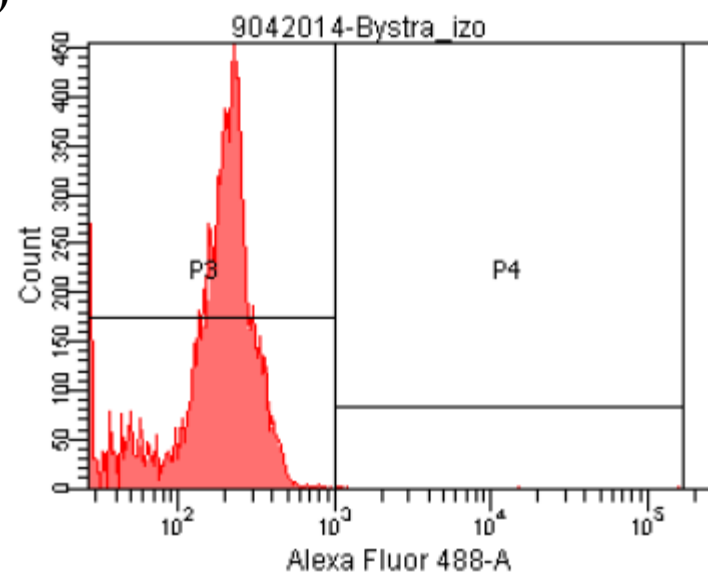

d)

| Tube: Bystra_izo |         |         |        |
|------------------|---------|---------|--------|
| Population       | #Events | %Parent | %Total |
| All Events       | 11,804  | ####    | 100.0  |
| P1               | 10,000  | 84.7    | 84.7   |
| P2               | 5       | 0.0     | 0.0    |
| P3               | 9,995   | 100.0   | 84.7   |
| P4               | 5       | 0.0     | 0.0    |

Supplement: Supplementary file 1 — Additional file 1: Representative results of flow cytometric analysis showing negative control of immunofluorescence staining of peripheral blood mononuclear cells (PBMC) from dog number 1 with ISACHC 2 class heart failure. PBMC were fixed, permeabilized and stained with secondary antibodies: chicken anti-mouse IgG conjugated with Alexa Fluor 488 (Molecular Probes, Life Technologies). The percentage of α-SMA positive cells is 0.0%; a) morphological flow cytometric parameters of analyzed cells gated on the basis of their size (forward scatter: FSC) and granularity (side scatter: SSC); b) cytogram of Alexa Fluor 488 positive cells (gate P2); c) histogram of Alexa Fluor 488 positive cells (gate P4); d) table showing population hierarchy of cells in cytograms a, b, and histogram c. (PDF 72 KB) [file 12864_2013_6215_MOESM1_ESM.pdf]

a)

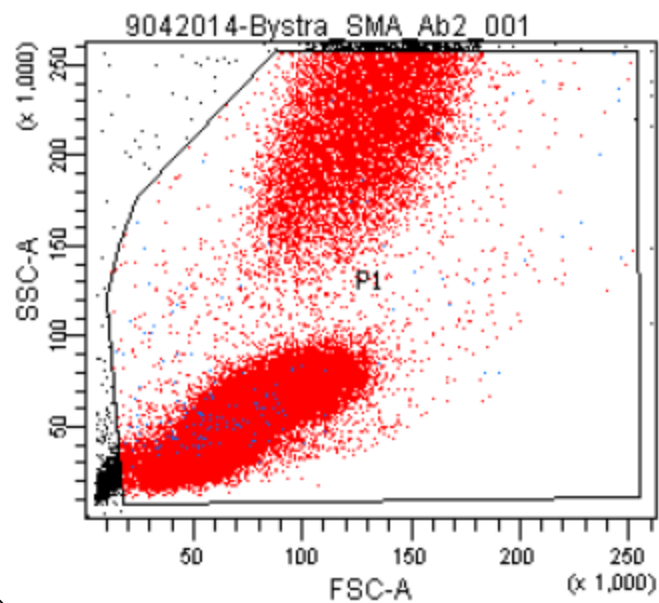

b)

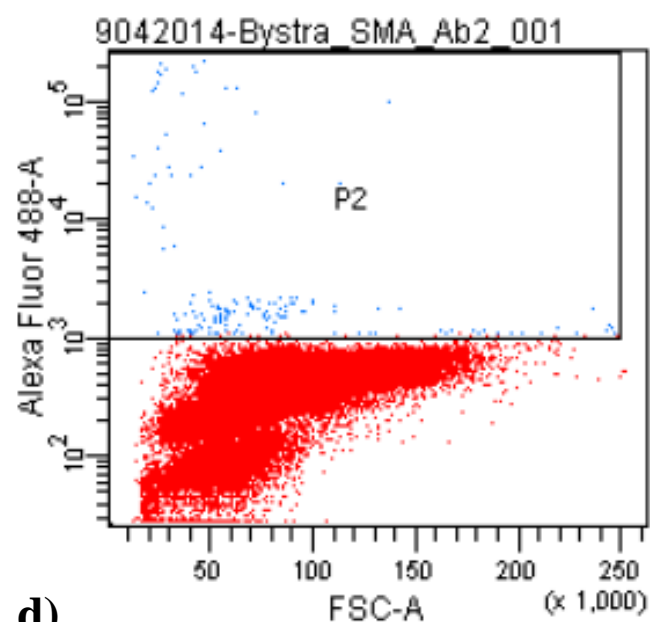

c)

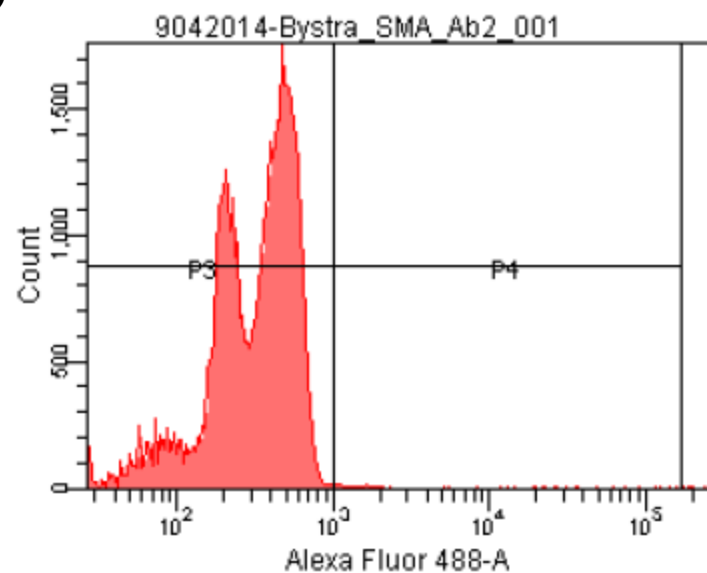

d)

Tube: Bystra\_SMA\_Ab2\_001

| Population | #Events | %Parent | %Total |
|------------|---------|---------|--------|
| All Events | 56,712  | ###     | 100.0  |
| P1         | 50,000  | 88.2    | 88.2   |
| P2         | 164     | 0.3     | 0.3    |
| P3         | 49,830  | 99.7    | 87.9   |
| P4         | 160     | 0.3     | 0.3    |

Supplement: Supplementary file 2 — Additional file 2: Representative results of flow cytometric analysis of peripheral blood mononuclear cells (PBMC) from dog number 1 with ISACHC 2 class heart failure, which were stained with antibodies against alpha smooth muscle actin (α-SMA). PBMC were fixed, permeabilized and stained with monoclonal mouse anti- α-SMA antibodies (clone 1A4, Dako, Denmark), followed by incubation with secondary antibodies: chicken anti-mouse IgG conjugated with Alexa Fluor 488 (Molecular Probes, Life Technologies). The percentage of α-SMA positive cells is around 0.3%; a) morphological flow cytometric parameters of analyzed cells gated on the basis of their size (forward scatter: FSC) and granularity (side scatter: SSC); b) cytogram showing α-SMA –positive cells gated in gate P2; c) histogram showing α-SMA –positive cells gated in gate P4; d) table showing population hierarchy of cells in cytograms a, b, and histogram c. (PDF 85 KB) [file 12864_2013_6215_MOESM2_ESM.pdf]
